# Local Similarity Search to Find Gene Indicators in Mitochondrial Genomes

**DOI:** 10.3390/biology3010220

**Published:** 2014-03-11

**Authors:** Ruby L. V. Moritz, Matthias Bernt, Martin Middendorf

**Affiliations:** Department of Computer Science, University of Leipzig, Postfach 100920, Leipzig D-04009, Germany; E-Mails: bernt@informatik.uni-leipzig.de (M.B.); middendorf@informatik.uni-leipzig.de (M.M.)

**Keywords:** suffix tree, conserved sequence, mitochondrial genomes, annotation

## Abstract

Given a set of nucleotide sequences we consider the problem of identifying conserved substrings occurring in homologous genes in a large number of sequences. The problem is solved by identifying certain nodes in a suffix tree containing all substrings occurring in the given nucleotide sequences. Due to the large size of the targeted data set, our approach employs a truncated version of suffix trees. Two methods for this task are introduced: (1) The annotation guided marker detection method uses gene annotations which might contain a moderate number of errors; (2) The probability based marker detection method determines sequences that appear significantly more often than expected. The approach is successfully applied to the mitochondrial nucleotide sequences, and the corresponding annotations that are available in RefSeq for 2989 metazoan species. We demonstrate that the approach finds appropriate substrings.

## 1. Introduction

Mitochondria are organelles that fulfill vital functions in eukaryotic cells. For example, they produce adenosine triphosphate which is an important carrier of chemical energy. Mitochondria are thought to have their evolutionary origin in *α*-proteobacteria which have been integrated into cells by endosymbiosis. Mitochondria inherited a genome (mitogenome) from their bacterial ancestors, which has been reduced dramatically in most lineages (see [1] for an overview).

The typical metazoan mitogenome is circular and usually comprises approximately 16.5 k nucleotides [2]. It comprises a nearly perfectly preserved gene content, consisting of 37 genes including 22 tRNAs, two rRNAs, and 13 protein coding genes. Compared to nuclear genomes it is very dense, i.e., besides the control region, which contains regulatory elements, non-coding regions are usually absent. With a few exceptions the genes encoded on a mitogenome consist of a single exon. Metazoan mitogenomes have a high AT content reaching values of 75% or more. A peculiar feature of metazoan mitogenomes is that the nucleotide frequencies differ (i) between the two strands (there is a guanine rich so called heavy strand); (ii) along the chromosomes [3]; (iii) as well as between species [4]. The order of the genes on mitogenomes has been subject to genome rearrangements that happened during evolution [2,5]. A recent, more detailed overview on metazoan mitogenetics can be found in [6].

Since mitogenomes are comparatively small and easy to sequence they are nowadays available for a wide variety of taxa. Currently (October 2013) nearly 3000 metazoan mitochondrial genomes are contained in NCBI RefSeq. This makes them a particularly interesting data set for phylogenetic studies among various taxonomic units [7]. They are also useful for studies in molecular ecology, biogeography, and their own molecular evolution that deviates vastly from that of the nuclear genome [8].

The NCBI GenBank and RefSeq data bases provide genome sequences and their corresponding annotations [9,10], i.e., in particular orientation and position of the genes. GenBank provides access to the data that has been submitted from labs all over the world through a common web front end. Based on GenBank, the aim of RefSeq is to provide a curated non redundant data set of higher quality. However, due to the multitude of data sources and methods used to annotate inconsistencies and errors are inevitable. The RefSeq annotations of metazoan mitochondrial genomes—which are the focus of this work—contain gene name inconsistencies, several cases of genes assigned to the wrong strand, genes that are missing in the annotation, and genes that are annotated superfluously [11,12]. Such problems cause problems for automated large scale analyses and might lead to confusion in studies that focus on selected taxa.

The MitoZoa database contains improved annotations that are obtained manually following a specified protocol [13]. Several tools for the automatic annotation of mitogenomes are available. DOGMA [14] uses tRNAscan [15] for the detection of tRNAs and BLAST for the remaining features. Automatic and high quality *de-novo* annotation of metazoan mitogenomes is possible with MITOS [12] which uses covariance model based ncRNA prediction based on MitFi [16] and a novel BLAST strategy for protein coding genes. Recently, MitoAnnotator [17], an annotation tool specialized for fish mitogenomes, was introduced that also used MitFi for the tRNAs and BLAST for the remaining features. Whereas DOGMA and MitoAnnotator need less than five minutes, MITOS currently needs more than one hour for the annotation of one mitogenome. Hence, a complete re-annotation of the available metazoan mitogenomes with state of the art tools needs between 10 days and half a year of computation time.

In this paper we introduce two efficient methods for computing marker sequences that can be used successfully as anchors of homologous subsequences in thousands of metazoan mitogenome sequences that both run within only a few hours.

To find homologous regions in two sequences the detection of very similar common substrings is often the first step, e.g., in the BLAST method [18]. The strictest kind of similarity is of course equality. The *suffix tree* data structure that stores the suffixes of sequences can be used to detect such identical substrings efficiently [19]. Suffix trees can be constructed in linear time and space using Ukkonen's algorithm [20]. *Generalized suffix trees* can store common substrings of multiple sequences [21]. For an overview on suffix trees and their applications see [22].

Generalized suffix trees are used for instance in MUMmer, which computes fast pairwise whole genome alignments using maximal unique matches (MUMs) as anchors [23]. Many state of the art whole genome alignment programs rely on (subsets of) MUMs, e.g., AVID employs them for building pairwise genome alignments [24], the approach of [25] uses MUMs required to appear in all input sequences, Mauve [26] uses MUMs of some minimal length, and [27] suggested to use only those MUMs as anchors which are an extension of a smaller exact match of a certain minimal length and do not contain an exact match appearing repeated in the same genome. A whole genome alignment is built from the anchors by chaining, i.e., determining a locally (e.g., [26]) or globally (e.g., [23]) co-linear subset of the anchors, and subsequent closing of the gaps between the anchors using dynamic programming sequence alignment. Thereby anchoring greatly reduces the computational cost.

Note that other methods for determining anchors are also available. LAGAN [28], for instance, allows a fixed number of mismatches in the anchors which are determined using a trie based k-mer index. Also in Murasaki [29] hashing of k-mers and gapped k-mers is used to identify anchors. Also rather different methods like FFT have been applied for detecting anchors, e.g., in MAFFT [30].

For large amounts of sequence data the linear space required by suffix trees becomes problematic. One approach to deal with this problem are *k-truncated suffix* trees that restrict the length of the stored substrings to *k*, i.e., the prefixes of length *k* of all suffixes [31,32]. In [33] this idea was extended to multiple sequences, i.e., *k*-truncated generalized suffix trees. This data structure detects common substring up to a length *k* efficiently.

In this work, we consider the following problem setting: For a set of given nucleotide sequences substrings that are common to some or all of these sequences should be determined as part of homologous sequences. For this problem, two methods are introduced that are based on *k*-truncated (generalized) suffix trees which are built for a given set of sequences. The methods determine the sought for common substrings as leaves in a further pruned version of these suffix trees.

The first method–called *Annotation Guided Marker Detection* method—extends the method described in [34] and decides the usability of a substring as anchor by checking if it appears nearly always within the same gene. To do so the method refers to gene annotations, the orientation, start and end positions of the genes, which are required as second input for each of the sequences. For the given annotations homology is assumed for genes with the same name and strand. Furthermore the given annotations potentially contain a moderate number of erroneous annotations. It was already shown in [34] that the preliminary version of the annotation guided marker detection method is very useful to automatically detect errors in given annotations. In particular, it was shown that different types of clear or likely errors could be detected in the RefSeq. For 203 annotated genes, for instance, a switch of the strand was proposed and for the tRNAs of Leucine and Serine (which both have two tRNA-coding genes denoted as *trnS1*, *trnS2* and *trnL1*, *trnL2*) a change of the numerals of the genes were proposed in 49 respectively 28 cases.

The second method - called *Probability Based Marker Detection* method - uses statistics that evaluate if the sequence occurs more often than expected in order to obtain the decision on the suitability of the sequence as anchor. A potential advantage of this method is that it does not rely on a given annotation.

The proposed methods may be used as an anchor or filtering method for future automatic *de-novo* annotation of large sets of DNA sequences. It is in particular promising for computationally more expensive approaches, e.g., [12]. They can also be used to detect errors in given annotations (as already done for a preliminary version of the first method in [34]).

In the next section the generalized suffix tree and the new methods for computing anchors of homologous subsequences are described. Results of their application to metazoan mitogenomes are presented in Section 4. Conclusions are given in Section 5.

## 2. Methods

The two marker detection methods that are introduced in this section use the generalized suffix tree data structure that is described in Subsection 2.1. The Annotation Guided Marker Detection method is introduced in Subsection 2.2 and the Probability Based Marker Detection method is introduced in Subsection 2.3.

### 2.1. Generalized Suffix Trees

Let *S* be a sequence over an alphabet Σ, |*S*| the length of *S*, and *S*^(^*^i^*^)^ with *i* ∈ [1 : |*S*| + 1] the suffix of *S* starting at position *i* including the empty suffix *S*^(|^*^S^*^|+1)^. The suffix tree *T* of a sequence *S* is a specific rooted tree whose edges are labeled with substrings of *S*$, for a symbol $ ∉ Σ. In the following we describe the main properties of the suffix tree (for a formal definition see, e.g., [22]). The *path label* of a node *v* is the concatenation of the labels of the edges along a path from the root to *v*. For every substring *S′* of *S*$ there exists exactly one subtree *T′* of *T* such that the nodes of *T′* are exactly the nodes that have *S′* as a prefix of their path label. Moreover, every prefix of a path label of a node in *T* is a substring of *S*$. Each node of the suffix tree is either a leaf or has at least two child nodes. The labels of two edges leaving the same node always start with different characters. The suffix tree of *S* has exactly |*S*| + 1 leaves such that for each *i* ∈ [1 : |*S|* + 1] exactly one of the leaves has the path label *S*^(^*^i^*^)^$.

Another feature of the suffix tree are the *suffix links* as introduced by [35] that allow for a linear time construction of the suffix tree. A suffix link is a link from node *v* with path label *αw*, *α* ∈ Σ and *w* ∈ Σ^+^, to node *v′* with path label *w*. Suffix links can be used to speed up search operations in the tree when a sequence is searched for several pattern-like markers in this case simultaneously. This kind of search is achieved in linear time in the size of sequence and independent of the number of searched patterns or pattern size.

The *generalized suffix tree* [21,36] can represent multiple sequences *S*_1_,…, *S_n_* over Σ at once. This can be achieved by building a suffix tree for *S*_1_$ and then extending the tree iteratively by integrating the sequences *S*_2_$, …, *S_n_*$. For a space efficient implementation of the generalized suffix tree as used in this paper the edge labels are not stored explicitly for each edge. Instead only the coordinates of the first occurrence of the edge label are stored. The coordinates of an occurrence are the positions of the first and last character of the occurrence in the concatenated sequence *S*_1_$*S*_2_$ … *S_n_*$. It is also possible to store additional information in the leaves, e.g., the number of occurrences of their path labels.

**Figure 1:**
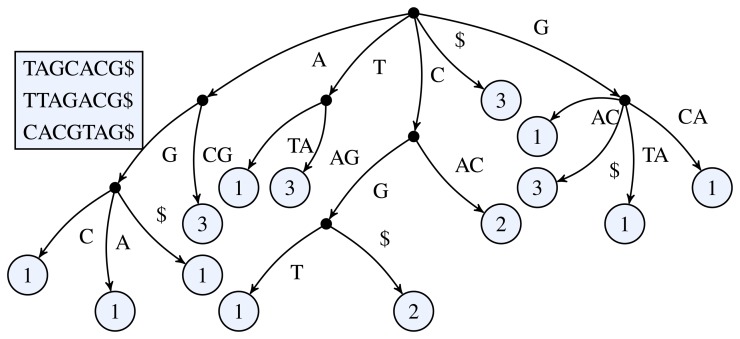
3-truncated generalized suffix tree of the sequences TAGCACG, TTAGACG, and CACGTAG. The black dots represent the inner nodes and the light circles the leaf nodes. The number inside a leaf node shows the number of occurrences of the leaf's path label in the input sequences.

In the following we consider a variation of the suffix tree restricting its depth to *k* as in [31,32] and its generalized version in [33]. The depth of this (generalized) suffix tree is measured as the maximal length of the path label of a leaf. If the generalized suffix tree for *S*_1_, …, *S_n_* is truncated at *k* it includes all substrings of *S*_1_$, …, *S_n_*$ up to length *k*. See Figure 1 for a simple example of a k-truncated generalized suffix tree.

For our application, i.e., thousands of mitogenomic sized genomes, the memory requirements of the k-truncated generalized suffix tree are suitable for moderately equipped computers. However, for larger applications (truncated) versions of more advanced data structures like one of the various versions of *enhanced suffix arrays* and *compressed suffix trees* might be necessary. These data structures need less memory than suffix trees, have different space-time tradeoffs, and are in practical applications generally superior to suffix trees (see, e.g., the recent papers [37,38]).

### 2.2. Annotation Guided Marker Detection

The annotation guided marker detection method was introduced in a previous preliminary version of this work [34]. The method assumes that nucleotide sequences *S*_1_, …, *S_n_* are each given with an annotation that provides the orientation, start, and end points for a set of genes occurring in that given sequence. As explained in the introduction the annotation might contain a limited amount of errors.

The annotation guided marker detection method uses an extension of the *k*-truncated generalized suffix tree that is called *annotation guided suffix tree*. In the annotation guided suffix tree for each gene *g* a score is stored with each leaf node *v* that measures the total overlap of all occurrences of the path label of *v* with gene *g* based on the provided annotation (Details of the score computation are described later). Note that, in [34] these scores were computed differently, i.e., as the number of occurrences of the path label whose start position is within the annotated gene. In the following we describe a more sophisticated scoring. Mind that genes on the forward strand are distinguished from genes on the reverse strand. Thus each gene *g* is handled as two separate genes *g*+ and *g*−.

The *k*-truncated generalized suffix tree *T* is constructed for the given sequences and their annotations such that whenever an occurrence of a path label of a leaf has been detected in the input sequences a comparison with the given annotation of the sequence is done. According to the sizes of the found overlaps with the annotated genes the score values of the leaf for the corresponding genes are increased. When all occurrences of the path labels of all leaves in the suffix tree have been detected and the score values of all leaves have been computed then the scores of the inner nodes are computed. This is done iteratively and bottom up such that for each inner node the score for a gene is computed as the sum of the corresponding scores of its child nodes. The score values in the suffix tree are used by our annotation guided marker detection method to determine which nodes have path labels that are suitable markers.

To define the score formally, let *n*(*v*) be the total number of occurrences of the path label of a leaf *v*. Let *occ_i_*(*v*) be the coordinates of the *i*th occurrence of *v*, *i* ∈ [1 : *n*(*v*)], i.e., the index of the sequence where *v* occurs and the positions of the first nucleotide and the last nucleotide of the occurrence. Let *ann_g_* be the annotation information for gene *g* over all available sequences. Then *overlap*(*occ_i_*(*v*), *ann_g_*) is defined as the total number of common nucleotide positions of the *i*th occurrence of *v* and the annotation of gene *g* on sequence *S* where *S* is the sequence of the *i*th occurrence of *v*. The score for a gene *g* and any node *v* is defined as:

(1) $$\text{score}\left(v,g\right)=\{\begin{array}{cc} \sum_{i=1}^{n(v)}\text{overlap}(occ_{i}(v),ann_{g}) & \text{if}\;v\;\text{is a leaf}\\ \sum_{v^{\prime }\text{is child of}\;v}score(v^{\prime },g) & \text{otherwise} \end{array}$$

Note that the score function does not count the overlap of the occurrences of the path label of an inner node with the given annotation of a gene *g*, because the path label of an inner node is shorter than the path labels of its child nodes. The score values that an inner node *v* receives from its child nodes rather count the respective overlaps of a genes annotation and all words of length *k* where the path label of *v* is a prefix.

We are not aware of a possibility to determine the genes overlaps with the path labels of an inner node in a time and memory efficient way because the coordinates of the occurrences are unknown in this subsequent step after the tree construction. However, the scores provide a sufficient approximation of the exact distribution of gene overlaps for the inner nodes.

For the marker detection the nodes whose path labels occur only once in the input sequences or are repetitive are dismissed in the further search for markers. A word *w* is a repetitive sequence if it contains a substring of the form *q^r^′^^* where *r′* is maximal and *r′* ≥ *r* for a parameter *r* ≥ 2 that is called the repeat threshold. See [39] for a recent analysis of repeats in metazoan mitochondrial genomes.

Algorithm 1 detects whether a word *w* is a repetitive string that contains a repeated string of up to *l* nucleotides in *O*(*l* · |*w*|) time. To this end it counts the occurrences of each word *q* with |*q*| ∈ [1 : *l*]. All words containing repeated substrings are considered unsuitable markers.

Algorithm 1 analyses word *w* for repeated substrings of length *j* + 1 in the outer for-loop starting in line 1. A window of length *j* + 1 is moved over word *w* from left to right starting in line 3. Whenever the first and last character in that window are unequal (*w_i_* ≠ *w*_*i*−*j*−1_ in line 4) the count is reset and the *offset* is set to the current window shift modulo *j*. The offset is used to check when the window was shifted by *j* + 1 positions without a single mismatch to increase the variable *count* which counts the number of repeats of word *w*_*i*−*j*−1_ … *w*_*i*−1_.



**Algorithm 1:** Repeat Detection applied on a word *w* = *w*_0_ …*w*_|*w*|−1_ with repeat threshold *r*
1**for**
*j* = 0 : *l* − 1 **do**2 *offset*←*j*;//modulo of start of repeat in *w*3 *count*←1;//current count of repetitions4 **for**
*i* = *j* + 1 : |*w*| − 1 **do**5  **if**
*w_i_* ≠ *w*_*i*−*j*−1_
**then**6   *count*←1;// substring w_*i*−*j*−1_ …*w*_*i*−1_ is not repeated7   *offset*← *i* mod (*j* + 1);8  *else if offset*= *i* mod (*j* + 1) **then**9   *count* ++;10   **if**
*count* > *r*
**then**11    **end** with: *w* contains a repeated substring;12**end** with: *w* contains no repeated substrings;


In a depth first search over the annotation guided suffix tree *T* nodes whose path labels are suitable as markers are detected. A node *v* is considered to be a suitable marker for a gene *g* if Inequality 2 holds and *g* = arg max*_g_ score*(*v, g*).

(2) $$\frac{\max_{g}\text{score}(v,g)}{\sum_{g}score(v,g)}>0.95$$

If a node *v* is found whose path label is suitable as marker the corresponding test is omitted for all nodes below of *v*. Since the path labels of the nodes below *v* have the path label of *v* as common prefix they cannot occur more frequently in the input sequences than the path label of their ancestor node *v*. As approximated by the score at least 95% of *v's* path label's occurrences in the given nucleotide sequences overlap with a single gene *g*. This information was gathered from *v's* child nodes. If there is at least one occurrence of *v's* path label overlapping with some gene *g′* different from *g* it is possible that some node below *v* might indicate an association of its path label with *g′*. However, we discard this information and consider it as an erroneous annotation.

Assume that a node *v* with path label *w* = *w′w″* is suitable as marker, i.e., it satisfies Inequality 2, and *v's* parent node *v′* has path label *w′* but does not satisfy Inequality 2. Then *m* = *w′u* where *u* is the first character of *w″*, i.e., the edge label from *v′* to *v*, is added to a set of candidate markers. Note that, *w′u* is the shortest prefix of *w* satisfying Inequality 2.

In a final step, the set of candidate markers is filtered such that no marker is suffix, prefix, or infix of another marker in the final set of markers. The filtering step is done as follows. Since no node *v* whose ancestor *v′* suffices Inequality 2 is tested for Inequality 2 it is ensured that no marker in the candidate set is prefix of another marker in this set. However, all markers that contain other markers as suffixes or infixes have to be removed. This is achieved by a search for all suffixes and infixes of a marker (see Algorithm 2). Of the remaining markers none is prefix, suffix or infix of any other marker.



**Algorithm 2:** Suffix Search applied on a node. For a node *v* with path label *w* = *w′w″* and parent node *v′* with path label *w′* the marker of a node is defined as *m* = *w′u* where *u* is the first character of *w″* (in case of the annotation guided suffix tree) or as *m* = *w′a* where *a* is the shortest prefix of *w″* such that *m* is significant (in case of the probability based suffix tree). If *w* does not suffice the requirements of the corresponding methods *m* is the empty word.
1*m* ← marker of this node2**for**
*i* ← 1 : |*m*| − 1 **do**3 *v* ← node with the shortest path label containing *m_i_*… *m*_|_*_m_*_|−1_ as prefix;4 *m′*
**←** marker of *v*5 **if**
*m′ exists and* |*m′*| ≤ |*m*| − *i* + 1 **then**6  **end** with: *m* contains another marker as infix7**end** with: *m* contains no other marker as infix


### 2.3. Probability Based Marker Detection

The marker detection method that is described in Subsection 2.2 relies on a given annotation. In this section we present the probability based marker detection method that detects markers using statistical tests and does not require annotation data. The tests are based on a Markov model of third degree derived from the given sequences. The Markov model is used to determine an expected number of occurrences for a word in a sequence whose {1,2,3,4}-mers occur with the same frequency as in the input sequences. These expected number of occurrences are compared with the actual number of occurrences.

The probability based marker detection method uses an extension of the *k*-truncated generalized suffix tree that is called *probability based suffix tree.* The construction of this tree is similar to the construction of the standard *k*-truncated generalized suffix tree but it needs a simple additional procedure to store for each leaf the total number of occurrences of its path label. To do so each leaf has a counter. During the construction of the tree all possible infixes of length *k* and suffixes shorter than *k* of the sequences *S*_1_$, …, *S_n_*$ are included into the tree. During the inclusion, the algorithm traverses the nodes of the tree until it reaches a leaf node. The leaf node increases its counter by one as the newly included infix or suffix equals the leaf's path label. When the inclusion process is finished and the counter values have been determined for all leaves, the counted number of occurrences are passed iteratively to the parent nodes. The number of occurrences of each inner node is computed as the sum of the values from the child nodes. The bottom up procedure takes linear run time.

When the probability based suffix tree has been constructed it can be used to detect markers. The path labels of the nodes are tested for their statistical significance by using their total number of occurrences as described in detail in the following. Similarly to the annotation guided marker detection method, this is done in a depth first search that does not search underneath nodes that proved to be significant.

Let *w* = *w*_0_*w*_1_ … *w*_|*w*|−1_ be the path label of some node *v* with *n*(*v*) occurrences. Let *α* ∈ Σ and *s* ∈ Σ^+^ then *P*(*s*, *a*) denotes the probability that *a* follows directly after s in a nucleotide sequence. For an empty string *s* we simply write *P*(*a*) denoting the probability of letter *a* occurring in a string. These probabilities are derived from a Markov model of third degree that is derived from the input sequences. We compute a probability for node *v's* path label to occur based on the probabilities from the Markov model by:

(3) $$P\left(w\right)=P\left(w_{0}\right)\cdot P\left(w_{0},w_{1}\right)\cdot P\left(w_{0}w_{1},w_{2}\right)\cdot \prod_{i=0}^{\left|w\right|-4}P\left(w_{i}w_{i+1}w_{i+2},w_{i+3}\right)$$

To identify whether a word can be used as a marker the statistical significance is derived from a χ^2^ significance test. The *χ*^2^ test takes the expected (*e_k_*) and the observed (*o_k_*) number of occurrences of *m* different categories that are a partition of the sample to compute a score $U_{m}^{2}$:

(4) $$U_{m}^{2}\sum_{k=1}^{m}\frac{{\left(o_{k}-e_{k}\right)}^{2}}{e_{k}}$$

In our case there are only two categories: (1) The word *w* starts at the current nucleotide or (2) not. The sample size *N* is the summed length of all input sequences. The expected and observed number of occurrences (*e*_1_, *o*_1_) of a word *w* that leads to the incoming edge, i.e., the path label or a prefix of the path label (including at least the path label of the parent), of node *v* and the complements of the number of occurrences (*e*_2_, *o*_2_) are derived from the following equations:

(5) $$o_{1}=n\left(v\right)$$

(6) $$o_{2}=N-o_{1}$$

(7) $$e_{1}=N\cdot P(w)$$

(8) $$e_{2}=N-e_{1}$$

This gives

(9) $$U_{2}^{2}=\frac{{\left(n\left(v\right)-N\cdot P\left(w\right)\right)}^{2}}{N\cdot P\left(w\right)}+\frac{{\left(N-n\left(v\right)-N\cdot \left(1-P\left(w\right)\right)\right)}^{2}}{N\cdot \left(1-P\left(w\right)\right)}$$

(10) $$=\frac{\left(n\left(v\right)-N\cdot P{\left(w\right)}^{2}\right)}{N\cdot P\left(w\right)\cdot \left(1-P\left(w\right)\right)}$$

The null hypothesis, i.e., *w* does occur randomly, is rejected when

(11) $$z<U_{2}^{2}$$

for some *z* > 0 such that the error of wrongly rejecting the null hypothesis (*α*) is small. In that case *w* occurs significantly more often than expected in random sequences based on the used Markov model.

If for the path label *w* of a node *v* Equation (11) is satisfied all nodes below *v* are dismissed, i.e., the significance of their path labels is not investigated. The reasoning for this is analogous to the corresponding reasoning for the annotation guided marker detection method. However, it is possible that a prefix of *w* would also satisfy Equation (11). Therefore, as we are only interested in the shortest significant string. Let *v′* be the parent node of *v* with path label *w′*. We test each string *w′w″* where *w″* is a prefix of the edge label from *v′* to *v* and take the shortest string as a candidate marker that satisfies Equation (11).

Equally to the guided marker detection method the set of candidate markers is reduced such that in the final set of markers no marker contains another marker as infix or suffix or contains a repeated substring.

Note, that with *probabilistic suffix trees* (PST) [40,41] a related method has been described. A PST is a pruned prefix tree of a set of sequences, i.e., the suffix tree of the reversed strings. Each node of the PST has assigned a probability distribution which gives the probabilities that one of the symbols of the outgoing edges follows the path label of the node. The probabilities are determined from observed frequencies of substrings of a given collection of strings. The trees are pruned such that only significant extensions are included. Hence a “PST is a compact representation of a variable order Markov chain which uses a suffix tree as index structure” [42]. Probabilistic suffix trees have been used successfully to discriminate coding from non coding regions in *E*. *coli* DNA [40] and for learning models of protein sequences [41]. In [43] PSTs have been used for clustering sequences by “learning” a separate PST for each cluster. In contrast to our method PSTs determine the probabilities from the observed occurrence frequencies of substrings of a given set of sequences. Whereas our method determines the probability of a substring to appear at random. We also like to refer the reader to the recent MoSDi software that contains several sequence analysis algorithms and can be used, for example, to count all q-grams in a text or compute motif statistics [44].

### 2.4. Classification of Markers

In the following we describe how to determine the *target gene* for each marker. The target gene of a marker is the gene this marker is associated with. Using the annotation guided method the target gene for every marker is clear. The probability based marker detection method, however, does not define a marker's target gene. To classify the markers, i.e., to detect their target genes, annotation information is necessary. Therefore, we assume for the following that an annotation is given. The annotation data is solely used to assign target genes to the markers not to determine the markers themselves.

Once the set of markers is determined all sequences are queried for these markers in a fast search over the suffix tree. The position of all occurrences of the markers are thus determined. In the next step these positions are compared with the gene annotations. The number of overlapping base pairs is counted for all gene annotations overlapping with a specific marker. Each marker gets a score for each gene that is defined by the summed size of overlaps with that gene. The gene with the highest score is finally chosen as the target gene of the marker.

### 2.5. Analysis of the Markers Quality

Having a set of markers with their respective target genes can be useful in various applications that are to a certain extend based on the detection of genes. One possible application is the detection and correction of errors in annotation databases. Other applications can be the inclusion into a *de-novo* annotation pipeline or the fast prediction of the presence or absence of genes.

The knowledge of the positions of markers in the nucleotide sequences and of the marker's target genes is used to produce a rough annotation of the sequences where subsequent overlapping markers with identical target genes are concatenated. The quality of the derived annotation is then compared with the original annotation.

The comparison of the rough annotation with the original annotation might show that some gene is placed in a different location or on the inverse strand. In these cases either our prediction, i.e., the rough annotation, or the original annotation is erroneous. It is not always clear which of the two possible ones is the case.

The comparison of the two annotations is done as in [12]. For each annotated fragment produced by our method a corresponding RefSeq annotation is defined. This annotation has to share the most positions with the predicted fragment and at least 25% of the total length of the fragment. These pairs of predicted fragments and RefSeq annotations are classified as (1) *equal* if both annotate the same gene and strand; (2) Δ± if they annotate the same gene but give different strands; (3) *false negatives* (FN) if a RefSeq annotation is unpaired; (4) *false positive* (FP) if a predicted fragment remains unpaired; and (5) *different* if the pair annotates different genes.

## 3. Experiments

To test our methods we use the annotations from the RefSeq data base (release 57) containing 2989 metazoan mitochondrial genomes [10]. Additional comparisons with the 2387 mitogenomes of the MitoZoa database [13] that are also contained in RefSeq have been carried out. The suffix tree was constructed with the original sequences and their reverse complements. The depth of the suffix tree is set to *k* = 30 as the vast majority of markers is shorter than 30 base pairs. Confronted with rather short words we set *l* = 4 and the repeat threshold *r* = 3. For the significance parameter we set *z* ∈ {25 000, 50 000, 25 000 000}. The algorithms are run on a PC with Six-Core AMD Opteron(tm) Processor 2435 with 32 GB RAM.

## 4. Results and Discussion

The annotation guided and probability based marker detection methods were run on the 2989 mitochondrial genome sequences contained in RefSeq and their reverse complements. Both approaches are very efficient (see Table 1), i.e., they run in less than 80 minutes.

**Table 1:** Runtime of one exemplary run.

	**Annotation Guided** (time in minutes)	**Probability Based (z = 50 k)** (time in minutes)
Construction phase	16	12
Marker detection phase	24	33
Detect marker on sequences	21	19
Classify marker	6	5

Σ	68	71

### 4.1. Structural Analysis

**Figure 2:**
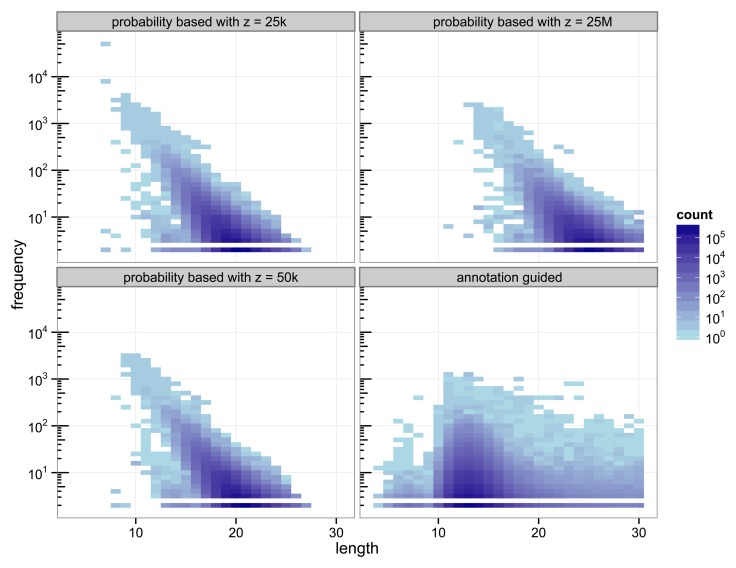
Heat map of the number of markers with a specific length and their number of occurrences.

The structure of the 30-truncated suffix trees, i.e., the annotation guided suffix tree and the probability based suffix tree, is identical for both methods. The number of nodes and leaves depends only on the chosen depth of the tree. For depth 30 the suffix tree consists of 133 786 893 nodes. The characteristics of the sequences that are chosen as markers differs strongly between the two methods (Figure 2).

With 3 511 781 markers the probability based method with *z* = 50 k detected more markers than the annotation guided method (3 119 929 markers). The results illustrate the trade off between marker length and number of occurrences. Clearly, short markers are more frequent than longer markers. The annotation guided method detected markers from 4 to 30 base pairs with an average marker length of 13.27 nucleotides and an average frequency of 6.31. The probability based method with *z* = 50 k detects markers with 8 to 27 nucleotides and an average length of 19.97 nucleotides. The markers frequency ranges from 2 to 3409 and is in average 6.61. Figure 2 indicates that the annotation guided method would have detected a large number of markers that are longer than 30 nucleotides if the depth of the suffix tree were allowed to be larger than 30. The figure also indicates that in this case it is unlikely that the probability based method would detect (many) markers with length greater than 30 (unless *z* is very large).

For the probability based method the length and frequency of the markers depends on the value chosen for z. Figure 2 shows the results for a small value of *z* = 25 k and a very large value of *z* = 25 M. With *z* = 25 k the marker length ranges from 7 to 27 nucleotides with an average of 19.45. The marker frequency ranges from 2 to 48 285 and is in average 6.93. Markers occurring more often than the number of sequences, i.e., the number of species, that are included in the analysis are qualitatively questionable as specifiers for certain genes because the genes usually occur at most once per genome. One of the markers with such a high frequency is the repeat (ATATATA) that is too short to be detected by Algorithm 1.

A very large value for z (25 M) reduces the frequencies of the detected markers to a range from 2 to 2642 and an average of 5.05. To satisfy Equation (11) with a large value for *z* the expected frequency of a marker has to be very small. Due to the underlying Markov model the expected frequency of a marker decreases with increasing length. Thus, only long sequences are declared as markers for a large value of *z*. For *z* = 25 M the obtained marker lengths range from 11 to 30 base pairs with an average of 24.63.

**Figure 3:**
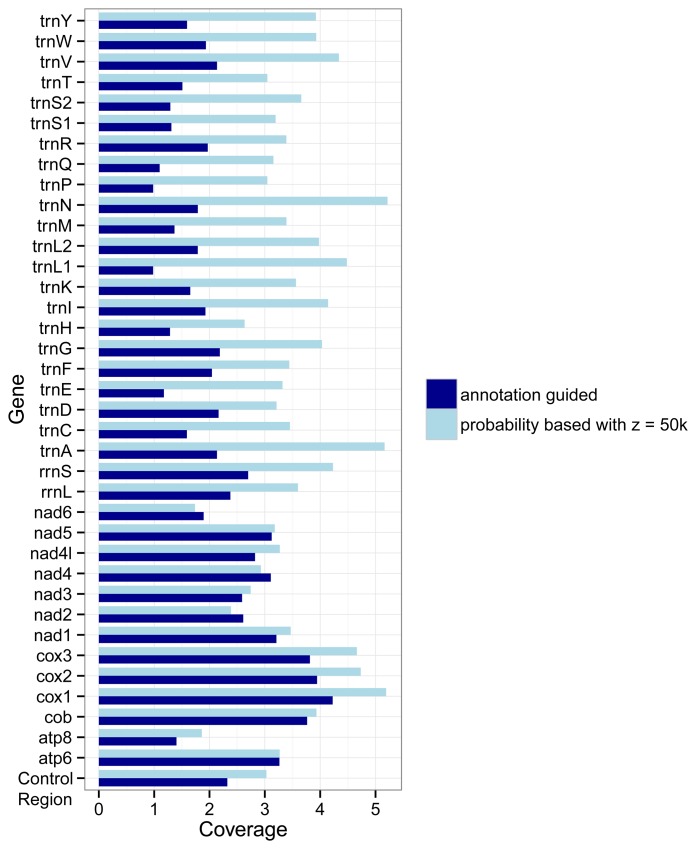
Coverage of genes by markers as detected with the annotation guided method and the probability based method. The coverage for a gene *g* is defined by the overlap of all markers with *g* as target gene with the annotations of *g* divided by the summed length of all regions annotated as *g* in the RefSeq.

The different approaches used by the two marker detection methods also show an effect on the number of markers for the different genes. Figure 3 shows how densely covered each gene is relative to its average length, i.e., its number of nucleotides. If the coverage of a gene *g* is *x* then on average each nucleotide of the gene overlaps with *x* markers for *g*. In most cases (the exceptions are *nad2*, *nad4*, and *nad6*) the probability based method covers the genes more densely than the annotation guided method. But differences between the two methods are larger for the tRNA genes and rRNA genes than for the protein coding genes.

**Figure 4:**
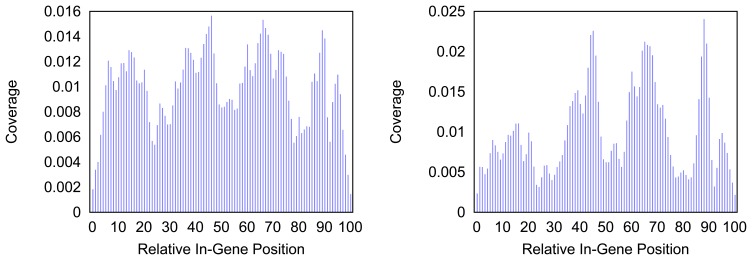
Density of the coverage with markers for gene *nad1* as detected by the annotation guided method (left) and the probability based method (right).

**Figure 5:**
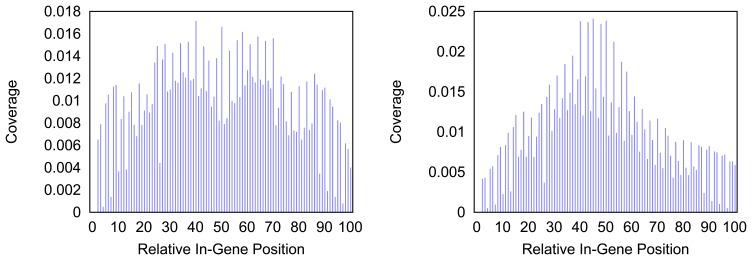
Density of the coverage with markers for gene *trnF* as detected by the annotation guided method (left) and the probability based method (right).

Figure 4 and Figure 5 give more insight on the distribution of markers over the different regions of a gene. In Figure 4 the coverage of the markers for the protein coding gene *nad1* (subunit 1 of the nicotinamide adenine dinucleotide, reduced dehydrogenase) is shown. Clearly there are regions in the gene that are more densely covered than others. Whether this is due to the conservation of specific functional or structural important regions in the gene is yet to be investigated. Both methods show the same distinctive peaks but the variation of the density is different. The slopes in the density produced by the probability based method are far steeper than the slopes in the comparative plot for the annotation guided method.

In Figure 5 the distribution of markers along the tRNA gene *trnF* are shown. Mind that the tRNA genes consist of less than a hundred nucleotides but the density for every hundredth part of a gene is measured. Thus a bar in the histogram represents less than one nucleotide and gives a rougher distribution than the respective distributions of longer genes. The distribution of the coverage does not immediately hint on the typical clover leaf structure of the tRNAs. However, in most tRNAs a central peak is visible where the anti codon is situated. In case of the *trnF* (Phenylalanine tRNA) the coverage with markers is generally higher towards the middle of the gene for both methods. However the annotation guided method shows a lower density in the very center of the gene than in the proximate areas around it.

### 4.2. Qualitative Analysis

The structural analysis of the methods gives insight on the basic characteristics of the markers. However, the quality of the markers cannot be derived by such means. In the following we compare the annotations given by the RefSeq database and the MitoZoa database with the rough annotations produced by the different marker detection methods.

Table 2 shows this comparison with the RefSeq database for the different types of genes for the classification according to Subsection 2.5. The data shows that the annotation guided method produces many false positives (FP) compared to the probability based method. A false positive is registered whenever there are markers for genes overlapping with unannotated regions in the RefSeq annotations. The probability based method has less false positives but more conflicts (different), where 25% of a prediction for a gene a overlaps with a region annotated as gene *b* ≠ *a* in the RefSeq annotations. The probability based method also switches the strandedness of the genes more often.

For protein coding genes it is quite obvious that the probability based method improves the number of false positives with an increased threshold. However, by reducing the number of false positives it also increases the number of false negatives (FN). A false negative (FN) is every gene of the annotation that does not overlap with any prediction produced by the algorithm. The number of correct predictions per gene decreases for stricter significance threshold values. The relative amount of false predictions is vastly reduced with large values for *z*, where *z* sets the significance threshold for the markers. The percentage of correct hits among all hits is the highest with *z* = 25 M, but the absolute number of correct hits per gene is the smallest compared to the other algorithms.

The results for the control region are similar to those of the protein coding genes. Aside from the probability based method with *z* = 25 k the quality of the markers is lower than those for the protein coding genes for the respective method, i.e., predictions are less correct.

For the rRNA genes the absence of false negatives is most striking. The remaining results are similar to those for the protein coding genes and control region. Among the 100 most frequent markers determined by the probability based method with *z* = 50 k 80 are associated with *rrnL* (the large ribosomal subunit) and 16 with *rrnS* (the small ribosomal subunit). Due to their near perfect conservation, i.e., 6 of the 8 most frequent markers and their reverse complements are associated with *rrnL* and occur in over 2850 sequences, each of these short sequences may serve as an indicator for the corresponding rRNA.

**Table 2:** Comparison of the annotations produced by the different methods (annotation guided and probability based with *z* = 25 k, 50 k, 25 M) and the ones given by the RefSeq database. The predictions proposed by the algorithms and the respective gene annotation that covers the prediction the most but at least 25% are classified as (1) *equal* if both annotate the same gene and strand; (2) Δ ± if they annotate the same gene but give different strands; (3) *false negatives* (FN) if a RefSeq annotation is unpaired; (4) *false positive* (FP) if a predicted fragment remains unpaired; and (5) *different* if the pair annotates different genes. The first column gives the number of predictions. The remaining columns present for each category the percentage wrt. all categories and the number normalized with the number of annotations in RefSeq is given. The respective best values are highlighted.

**#Predictions**		**Equal**		**Δ±**		**FN**		**FP**		**Different**	
**Proteins:** 38 733 annotations in RefSeq											
A. G.	348,514	62%	5.72	0%	0.00	0%	0.00	35%	3.21	2%	0.22
25 k	309,737	63%	5.05	2%	0.14	0%	0.03	2%	0.13	33%	2.68
50 k	204,415	92%	4.90	0%	0.03	1%	0.04	0%	0.02	6%	0.32
25 M	167,490	95%	4.18	0%	0.00	2%	0.10	0%	0.01	3%	0.13

**Control Region**: 3153 annotations in RefSeq											
A. G.	39,857	59%	7.49	0%	0.00	1%	0.14	38%	4.88	2%	0.26
25 k	23,839	63%	4.83	6%	0.48	1%	0.06	6%	0.48	23%	1.77
50 k	18,165	79%	4.63	6%	0.35	2%	0.13	6%	0.38	7%	0.41
25 M	13,228	81%	3.54	2%	0.09	4%	0.17	6%	0.28	6%	0.28

**rRNAs:** 5967 annotations in RefSeq											
A. G.	92,807	69%	10.79	0%	0.00	0%	0.00	29%	4.44	2%	0.31
25 k	87,034	57%%	8.35	5%	0.66	0%	0.00	2%	0.27	36%	5.30
50 k	60,131	81%	8.14	1%	0.12	0%	0.00	2%	0.16	16%	1.65
25 M	44,616	94%	7.04	1%	0.09	0%	0.00	1%	0.07	4%	0.28

**tRNAs:** 63 972 annotations in RefSeq															
A. G.	105,313	74%	1.27	0%	0.00	4%	0.07	12%	0.21	10%	0.16
25 k	101,554	56%	0.96	3%	0.06	7%	0.11	1%	0.01	33%	0.56
50 k	73,926	71%	0.91	1%	0.01	10%	0.13	1%	0.01	18%	0.23
25 M	63,524	63%	0.78	3%	0.04	20%	0.25	0%	0.00	14%	0.17

The tRNA genes are generally shorter than the other genes considered. This complicates the detection of markers as the total number of markers is lower for these genes. The annotations for the tRNAs produced by the marker detection methods miss many genes (FN) or lie in regions of other genes based on the annotations provided by the RefSeq data base. The annotation guided method collected the highest number of correct predictions per gene for the tRNAs compared to the probability based method.

Bear in mind that the annotations provided by the RefSeq may contain some errors possibly distorting these results. In an additional analysis we compared our results that were produced using the RefSeq annotations with the curated annotations provided by the MitoZoa database (see Table 3).

**Table 3:** Comparison of the annotations produced by the different methods (annotation guided and probability based with *z* = 25 k, 50 k, 25 M) and the ones given by the MitoZoa database. The predictions proposed by the algorithms and the respective gene annotation that covers the prediction the most but at least 25% are classified as (1) *equal* if both annotate the same gene and strand; (2) Δ± if they annotate the same gene but give different strands; (3) *false negatives* (FN) if a RefSeq annotation is unpaired; (4) *false positive* (FP) if a predicted fragment remains unpaired; and (5) *different* if the pair annotates different genes. The first column gives the number of predictions. The remaining columns present for each category the percentage wrt. all categories and the number normalized with the number of annotations in MitoZoa is given.The respective best values are highlighted.

**#Predictions**		**Equal**		**Δ±**		**FN**		**FP**		**Different**	
**Proteins:** 31 112 annotations in MitoZoa											
A. G.	272,206	65%	5.69	0%	0.00	0%	0.00	31%	2.74	4%	0.31
25 k	248,338	63%	5.07	2%	0.13	0%	0.03	2%	0.17	33%	2.61
50 k	164,209	92%	4.92	0%	0.03	1%	0.04	0%	0.02	6%	0.32
25 M	134572	95%	4.20	0%	0.00	2%	0.10	0%	0.01	3%	0.12

**Control Region:** 2486 annotations in MitoZoa											
A. G.	31,097	58%	7.35	0%	0.02	1%	0.15	37%	4.75	3%	0.40
25 k	19,408	62%	4.86	5%	0.43	1%	0.06	9%	0.68	23%	1.84
50 k	14,852	76%	4.63	5%	0.31	2%	0.12	9%	0.57	7%	0.46
25 M	10,699	79%	3.51	1%	0.07	3%	0.15	9%	0.41	7%	0.31

**rRNAs:** 5785 annotations in MitoZoa											
A. G.	71,730	71% 1	0.69	0%	0.06	0%	0.00	25%	3.80	3%	0.44
25 k	69,967	58%	8.45	4%	0.63	0%	0.00	1%	0.17	37%	5.38
50 k	48,330	82%	8.24	1%	0.10	0%	0.00	1%	0.09	16%	1.66
25 M	35,830	95%	7.09	1%	0.06	0%	0.00	0%	0.04	4%	0.30

**tRNAs:** 51 575 annotations in MitoZoa											
A. G.	83,742	75%	1.27	0%	0.00	4%	0.07	11%	0.18	10%	0.17
25 k	81,302	57%	0.96	3%	0.06	7%	0.11	1%	0.01	32%	0.54
50 k	59,229	72%	0.92	0%	0.01	10%	0.13	1%	0.01	17%	0.22
25 M	50,681	63%	0.78	3%	0.04	21%	0.26	0%	0.00	13%	0.16

The comparison gives similar values to those shown in Table 2. The absolute number of predictions and annotated genes are smaller because the subset of annotated mitogenome sequences that are available in both MitoZoa and RefSeq (2387) are less than the annotations provided by RefSeq (2989).

Aside from the annotations for the control region the comparison of the annotations provided by our methods score more true positives than in the comparison with the RefSeq. There are equally or less strand differences in the comparison with MitoZoa than with the RefSeq. Only in two cases (tRNA and control region, probability based with *z* = 25 M) there are more false negatives. The number of false positives is equal or lower except for the control region where an increase for the probability based methods is registered. The number of differently predicted genes is higher in three cases, lower in four cases (especially tRNAs), and similar otherwise.

In general, the MitoZoa annotations are slightly better fitting to the annotations produced by the annotation guided and probability based method, although the annotations provided by the RefSeq were used to produce those annotations.

However, the control region has a higher rate of false positives and a lower rate of true positives in comparison with the MitoZoa than with the RefSeq. While the RefSeq contains annotations for the control region for 2409 species the MitoZoa provides such annotations for only 453 species.

These results show that the proposed methods are robust towards erroneous annotations. We suppose the MitoZoa database has less erroneous annotations than the RefSeq database that was used by our methods to generate new rough annotations. These annotations show a higher resemblance with the hand curated data from the MitoZoa, thus correcting errors that are contained in the RefSeq data. However the annotation of the control region is often missing or uncertain leading to more false positives and less true positives.

For the probability based marker detection method (with *z* = 50 *k*) a large number (38 369) of predictions fall into the category different, i.e., the prediction overlaps most with a different feature of the RefSeq annotation. In 9971 of these cases the predicted gene and the annotated gene are adjacent in the genome. This large number can be explained by the fact that the method determines conserved sequences. If gene boundaries are conserved our methods will find conserved sequences spanning the boundary of two genes. In mitogenomes the same genes are often adjacent. In particular the mitogenomes of most chordates and many insects share a common gene order. Furthermore, mitochondrial genes are tightly packed on the mitogenome, i.e., there is mostly no or extremely small non-coding regions between genes or they might even overlap by a few nucleotides [16].

The control region harbors most of the signals for replication and transcription of the metazoan mitogenome [45], see also review in [6]. The control region is fast evolving and its characteristics differ between taxa [46,47] and thereby annotation depends on the availability of closely related mitogenomes with an annotated control region. This holds in particular if the annotation method solely relies on exact matches. The status of the annotation of the control region differs strongly between taxa. While RefSeq provides annotation for 93% of the deuterostome mitogenomes, for only 62% of protostome mitogenomes an annotation is provided. Within Protostomia even more extreme differences are found: Ecdysozoa 78%, Lophotrochozoa 12%. Furthermore, the sparsity of available annotation coincides with a small number of sampled mitogenomes: 2024 Deuterostomia, 807 Protostomia, 622 Ecdysozoa, and 185 Lophotrochozoa. This is clearly reflected in the predictions made with our methods. The probability based marker detection method (with *z* = 50 *k*) finds a correct marker, for only 67% of the annotated control regions in protostomia but in 98% for the Deuterostomia. Within the Protostomia consistent results are found. For the very sparsely sampled and annotated Lophotrochozoa 39% of the predictions match the RefSeq annotations and for the better sampled and annotated Ecdysozoa 68% do match.

Prediction quality differs between taxa. The number of predictions of the probability based method (with *z* = 50 *k*) that are classified as different is on average 15.53 for Deuterostomia and only 6.55 for Protostomia (Ecdysozoa 6.16 and Lophotrochozoa 7.87).

For a comparison with a state of the art tool that is reporting exact matches we used MUMmer [23] (Version 3.23). MUMmer constructs a suffix tree for a set of reference sequences which is used to extract exact pairwise matches of these sequences with a set of query sequences. No uniqueness of the reported pairwise matches is required (i.e., parameter -maxmatch is used) since otherwise less than 216 750 pairwise matches are reported. We used the set of all mitogenome sequences from RefSeq 57 as reference and query in order to get a comparison data set. Since our methods do not report pairwise matches but sets of exact matches a direct comparison was not possible. Therefore we constructed for the results of the probability based marker detection with *z* = 50 *k* for each marker the set of all pairs of subsequences that are represented by the marker. In the following a comparison of these two sets of pairwise matches is presented.

MUMmer computed in 192 s a set of 0.1 × 10^9^ pairwise matches of average length 28 nt. The markers computed by our method correspond to a much larger set of more than 1.5 × 10^9^ pairwise matches of average length 12 nt. The differences are clearly caused by the fact that MUMmer computes maximal matches for pairs of sequences which are at least as long as matches for larger sets of sequences as computed by our method. Furthermore our method computes matches that are not longer than necessary which gives a larger number of pairwise matches that are shorter on average. For a comparison of the quality of the reported matches we determined for each pairwise hit the gene that has the largest overlap with the reference and query sequence of the match. The orientation of the genes is ignored here. MUMmer found the same gene in only 61% of the cases whereas the probability based marker detection finds a pairwise match of equal genes in 97% of the cases.

## 5. Conclusions

We have introduced two methods for determining substrings of homologous sequences from large sets of sequences. These sequences are determined in a *k*-truncated generalized suffix tree. The first method, called annotation guided marker detection method, uses additional gene annotation data which may include a small fraction of errors. The second method, called probability based marker detection method, is independent of annotation data and determines the substrings by testing their statistical significance using expected frequencies derived from a Markov model. Both methods have been implemented and tested for the available metazoan mitochondrial genome sequences in the RefSeq database. The probability based marker detection method has been evaluated for multiple significance thresholds. For higher thresholds a smaller number of substrings are reported which are on average longer and appear less frequent. Compared with the probability based marker detection method using a medium significance threshold the annotation guided method returns a similar number of markers which appear less frequent and are much shorter on average. A comparison of the annotations with reference annotations shows that both approaches can be used to obtain conserved sequences in homologous genes.

The proposed suffix tree based marker detection methods show great potential in the detection of short sequences that can be used for the detection of genes. The result of the methods can be used to improve approaches annotating mitochondrial genomes or to detect and correct contained erroneous annotations. The probability based marker detection method is solely based on the nucleotide sequences and no knowledge about the origin of the chosen sequences aside of their structure and frequency. Although the annotation guided method takes more information into account it shows no improvement in the results.

Compared to methods using k-mers or truncated suffix trees of fixed depth our approach is more suitable for sequences with high variation in the nucleotide frequencies, e.g., mitochondrial genomes. The reason is that these methods can not accommodate for the expected variation of the probabilities. Homologous conserved sequences might eventually be extracted as highly conserved regions from a multiple whole genome alignment of the given sequences. For metazoan mitogenomes this is not possible for contemporary methods for the following reasons: (1) huge number of sequences renders this computationally difficult; (2) large number of genome rearrangements excluded all methods using a global chaining of anchors; and (3) due to the large taxonomic range the sequences are highly divergent which makes exact matches over all sequences, that are the base of most aligning programs, basically impossible. The substrings identified by our method might be useful for constructing a whole genome alignment if an efficient local chaining algorithm for sets of anchors appearing in a subset of the sequences becomes available.
